# Persistent disease burden despite advanced therapies in inflammatory bowel disease: a real-world patient-reported survey from Greece

**DOI:** 10.3389/fimmu.2026.1866461

**Published:** 2026-07-10

**Authors:** Charalampos Tzanetakos, Vasiliki-Rafaela Vakouftsi, George Mavridoglou, Andriani Angelopoulou, George Gourzoulidis

**Affiliations:** 1 Health Through Evidence, Athens, Greece; 2 Hellenic Society of Crohn’s disease’s and Ulcerative Colitis’ patients (HELLESCC), Athens, Greece; 3 University of Peloponnese, Kalamata, Greece

**Keywords:** advanced therapies, Crohn’s disease, disease burden, patient-reported outcomes, ulcerative colitis, unmet medical needs

## Abstract

**Objectives:**

Despite therapeutic advances, inflammatory bowel disease (IBD) remains a lifelong debilitating disorder with substantial burden. The study aimed to investigate disease burden and unmet needs in IBD patients receiving advanced therapies in Greece.

**Methods:**

Between October 2023 and January 2024, adult CD and UC patients who were members of Hellenic Society of Crohn’s disease’s and Ulcerative Colitis’ patients (HELLESCC) and receiving advanced therapies completed a structured self-reported questionnaire. The survey collected data on sociodemographic characteristics, smoking status, comorbidities, disease activity, disease characteristics, current medications, and patient-reported outcomes (PROs; Short Inflammatory Bowel Disease Questionnaire [SIBDQ], Work Productivity and Activity Impairment [WPAI], Patient Health Questionnaire-9 [PHQ-9], treatment satisfaction and adherence). Both univariate and multivariate logistic regression analyses were performed to determine associated factors.

**Results:**

Among 287 patients with IBD (201 with CD, 86 with UC) receiving advanced therapies, 57.1% had active disease. Overall, 76.4% reported impaired QoL, 30.3% work productivity loss, and 38.4% activity impairment, while nearly half exhibited moderate-to-severe depressive symptoms. Notably, 39.1% expressed dissatisfaction with their advanced therapy, while 10.9% reported reduced adherence. No significant differences were observed between CD and UC patients across PROs, except for adherence. Higher disease activity was consistently associated with worse QoL, greater work impairment, increased depressive symptoms, and lower treatment satisfaction.

**Conclusion:**

Despite advanced therapies, IBD burden remains substantial among Greek patients. A considerable proportion experience poor QoL, increased work impairment, significant depressive symptoms, and persistent disease activity. Marked treatment dissatisfaction, reported by four out of ten patients, highlights an unmet need for more effective management strategies.

## 1 Introduction

Inflammatory bowel disease (IBD), mainly represented by Crohn’s disease (CD) and ulcerative colitis (UC), is a chronic debilitating disorder characterized by immune-mediated inflammation of the gastrointestinal tract (1). Both CD and UC follow a progressive clinical course, defined by alternating periods of relapse and remission, and present a broad spectrum of extraintestinal, systemic manifestations in addition to intestinal damage (2, 3).

Over recent decades, the therapeutic landscape for IBD has evolved considerably (4). The advent of biologic therapies, such as anti-tumor necrosis factor (TNF), anti-integrin and anti-interleukin (IL)-12/23 agents, along with newer small-molecule drugs including Janus Kinase (JAK) inhibitors and Sphingosine-1-Phosphate (S1P) receptor modulators, has enabled more targeted treatment approaches and improved clinical outcomes (5). However, clinical effectiveness remains modest with persistent significant unmet needs, including a “therapeutic ceiling” in treatment response, high rates of disease relapse and treatment-related toxicity (5). This is also reflected in real-world evidence demonstrating suboptimal persistence with biologic therapies, often driven by dissatisfaction with treatment response or adverse events (6). Notably, fewer than half of IBD patients remain on their initial biologic therapy after one year (48.48% in CD and 44.78% in UC) (6). As a result, many patients continue to experience a considerable disease burden, characterized by ongoing symptoms, increased pain and fatigue, and impaired health-related quality of life (HRQoL) (5, 7).

Growing recognition that objective measures of disease activity may not adequately reflect the multifaceted nature of IBD has led to the increasing integration of patient-reported outcomes (PROs) into disease assessment (8–10). Consequently, PROs are now recognized as essential endpoints in both clinical trials and clinical practice of IBD (8, 9, 11). Validated questionnaires commonly used to quantify these outcomes include the Short Inflammatory Bowel Disease Questionnaire (SIBDQ) for disease-specific quality of life (QoL) (12–16), the Work Productivity and Activity Impairment (WPAI) questionnaire for work-related outcomes (17–19), and the Patient Health Questionnaire-9 (PHQ-9) for depressive symptoms (20, 21).

Global real-world evidence incorporating PROs highlight that despite advanced therapies, IBD is characterized by persistent symptoms, flares, and diminished QoL (7, 22–24). Consistent with this, data from Greece indicate that IBD patients continue to experience impaired QoL, work impairment, depression, and ongoing disease activity (25, 26). Although UC patients report improved satisfaction with physician care and disease management, communication gaps persist, particularly around QoL and psychosocial concerns (27), while CD patients appear to face greater limitations in daily and social functioning (28). However, Greek data remain limited, particularly in the context of patients receiving advanced therapies, and may not fully capture the multidimensional burden of IBD in this treatment setting.

In this context, the present study aimed to evaluate the ongoing burden and unmet medical needs of IBD in patients who receive advanced therapies in Greece. For this purpose, a structured questionnaire employing a comprehensive set of validated PRO instruments was distributed to members of the Greek national IBD patients’ association. Associations between demographic and disease-related factors, and PROs were also investigated.

## 2 Materials and methods

### 2.1 Study design and population

From October 2023 to January 2024, a cross-sectional survey was conducted in collaboration with Hellenic Society of Crohn’s disease and Ulcerative Colitis’ patients (HELLESCC) (25, 26). Eligible participants were adults (age≥18 years) diagnosed with UC or CD, members of this Greek patient association. The questionnaire was distributed as a link via email or phone. Recruitment was conducted by HELLESCC staff without collecting participants’ personal data. Prior to participation, all participants were informed about the purpose of the study and were asked to provide their consent. Survey participation was entirely voluntary, and participants had the right to withdraw at any time. All collected data were kept anonymous and handled confidentially.

### 2.2 Questionnaire and variables

The questionnaire was developed in Greek (25, 26). Collected variables included sociodemographic characteristics, smoking habits, history of comorbidities, disease characteristics, disease activity, current medications, and PROs (SIBDQ, WPAI, PHQ-9, treatment satisfaction, and treatment adherence). Disease activity in UC was evaluated using the Simple Clinical Colitis Activity Index (SCCAI) index score (29) and classes were defined as follows: remission (0–2), mild (3–5), moderate (6–11) and severe (>11) (30, 31). For CD, disease activity was measured with the Harvey-Bradshaw Index (HBI), defining remission (0-4), mild (5-7), moderate (8-16) and severe (>16) (32). All available prescription medications at the time of survey were recorded. These included advanced therapies, such as tumor necrosis factor inhibitors (TNFi), an integrin α4 inhibitor, an interleukin-12/23 inhibitor (IL-12/23i), and Janus kinase inhibitors (JAKi) as well as non-advanced therapies such as 5-aminosalicylic acids (5-ASAs), corticosteroids, immunosuppressants and antibiotics.

The questionnaire included validated measures to evaluate the following PROs: QoL (SIBDQ), work productivity (WPAI) and psychological burden (PHQ-9) (25, 26). The SIBDQ scores range from 10 to 70 (16, 33), with QoL impairment classified as mild, moderate or severe for SIBDQ scores of 60-70, 45–59 and 10-44, respectively (15, 16, 34). The SIBDQ scores below 60 were considered indicative of moderate-to-severe QoL impairment and scores of 60 or higher were categorized as normal QoL. The WPAI scores (0-100%) measure absenteeism (work time missed), presenteeism (impairment at work), work productivity loss (overall work impairment/absenteeism plus presenteeism), and activity impairment (17, 35). Similar to previous studies, WPAI scores were categorized as mild (0-19%), moderate (20-49%), and severe (≥50%) (25, 26). PHQ-9 scores range from 0 to 27, with increasing values showing greater depressive symptom severity. The threshold score of 10 or above corresponds to moderate-to-severe depressive symptoms, potentially indicative of clinically relevant depression. Severity categories were defined as follows: 0-4 = minimal or none; 5-9 = mild; 10-14 = moderate; 15-19 = moderately severe; 20-27 = severe) (21, 36, 37).

Treatment satisfaction was evaluated with a study-specific, 5-point Likert scale question capturing participants’ level of satisfaction with their current treatment (not at all; little; quite; a lot; very much satisfied) (25, 26). Participants reporting lower satisfaction or dissatisfaction, defined as responses of “not at all,” “little,” or “quite satisfied”, were also asked to specify the reasons underlying their dissatisfaction. A study-specific, 5-point Likert scale question was also used to assess treatment adherence (I follow my treatment regularly; there are few times I forget to/I do not take my treatment; sometimes I forget to/I do not take my treatment; many times, I forget to/I do not take my treatment; I never take my treatment), and among participants reporting suboptimal adherence, further data were collected on factors contributing to non-adherence (25, 26).

### 2.3 Statistical analysis

Categorical variables were summarized using frequencies (n) and percentages (%), while continuous variables using means and standard deviations (SD). Sociodemographic and clinical variables, and PROs (SIBDQ, WPAI-UC, PHQ-9, treatment satisfaction and treatment adherence) were described by disease type (CD or UC). The association of disease type with sociodemographic and clinical factors was investigated with the Pearson’s χ2 test for categorical variables and with the Mann-Whitney test for continuous variables. The mean or proportional differences between CD and UC groups and their corresponding 95% confidence intervals (CIs) were also calculated. CIs enhance clinical interpretation by providing a plausible range of values in the actual units of measurement, along with the direction and strength of the effect (38). It is important to note that the outcome comparisons are cross-sectional and should not be interpreted as differences in response to treatment. Relationships between PROs and disease activity were quantified using the Spearman’s correlation coefficient (SCC). Sociodemographic and clinical factors associated with PROs were analyzed at both bivariate and multivariate levels using logistic regression. Factors with a p-value < 0.15 in bivariate analyses were included in a multivariate logistic regression model with stepwise selection. Effect sizes are presented as odds ratios (OR) with 95% CIs. Statistical significance was defined as p < 0.05. Data cleaning, data manipulation and data analysis were conducted using the statistical software IBM SPSS Statistics 29.0.

## 3 Results

### 3.1 Participants characteristics

The questionnaire was sent to 1.334 patients and returned by 472 [participation rate: 35.4%] (**Supplementary Figure 1**). A total of 287 patients were receiving advanced therapies and constituted the study population (51 patients who were not receiving any IBD-related drug therapy and 134 patients who were not receiving advanced therapy were excluded from the analysis). The mean age [± SD] was 41.8 [10.4], 57.8% were female, and most participants (54.9%) were in paid employment. The mean age of diagnosis was 30.5 [10.7] and the mean disease duration was 11.3 [7.4]. The mean time from symptom onset to diagnosis was 14.4 months [18.2]. During the last 12 months, the mean number [SD] of gastroenterologist visits was 4.3 [8.3] and 17.7% of patients had at least one hospitalization. A total of 131 patients (65.2%) had at least one comorbidity (**Table 1**), with arthritis (38.9%) and depression (29%) being the most frequently reported (**Supplementary Table 1**).

**Table 1:** Characteristics of the study population.

Characteristics	Total (n= 287)	CD (n=201)	UC (n=86)	Difference (95% CI)^a^	p-value^b^
Age, years					
Mean [SD]	41.8 (10.4)	41.8 (10.1)	41.9 (11.2)	-0.1 (-2.7-2.5)	0.939
Gender, n (%)	n=287	n=201	n=86		
Male	121 (42.2%)	81 (40.3%)	40 (46.5)	-6.2% (-18.6% - 6.2%)	0.199
BMI, n (%)	n=287	n=201	n=86		
Underweight (<18.5)	10 (3.5%)	9 (4.5%)	1 (1.2%)	3.3% (-1.7% - 7%)	0.380
Normal (18.5–25)	104 (36.2%)	71 (35.3%)	33 (38.4%)	-3% (-15.3% - 8.0%)	
Overweight (25–30)	116 (40.4%)	84 (41%)	32 (37.2%)	4.6% (-7.8% - 16.6%)	
Obese (≥30)	57 (19.9%)	37 (18.4%)	20 (23.3%)	-4.8% (-15.5% - 5.3%)	
Residence, n (%)	n=287	n=201	n=86		
Urban area (>10.000 residents)	243 (84.7%)	172 (85.6%)	71 (82.6%)	3.0% (-6.0%, 12.8%)	0.592
Family status, n (%)	n=206	n=141	n=65		
Married	107 (51.9%)	71 (50.4%)	36 (55.4%)	-5% (-19.3% - 9.6%)	0.55
Socioeconomic status, n (%)	n=206	n=141	n=65		
In paid employment^§^	113 (54.9%)	76 (53.9%)	37 (56.9%)	-3.0% (-17.3% -11.5%)	0.764
Education level, n (%)	n=206	n=141	n=65		
Bachelor degree or more	115 (55.8%)	73 (51.8%)	42 (64.6%)	-12.8% (-26.5% - 1.7%)	0.057
Smoker, n (%)	n=287	n=201	n=86		
Current smoker	101 (35.2%)	83 (41.3%)	18 (20.9%)	20.4% (8.8% - 30.7%)	0.001
Age at diagnosis, years	n=287	n=201	n=86		
Age at diagnosis, Mean [SD]	30.5 (10.7)	30.5 (10.5)	30.4 (11.6)	-0.1 (-2.7-2.5)	0.939
Disease duration, years	n=287	n=201	n=86		
Disease duration, Mean [SD]	11.3 (7.4)	11.3 (7.6)	11.5 (6.9)	-0.2 (-2.1-1.7)	0.824
Time from symptoms’ onset to diagnosis, months	n=287	n=201	n=86		
Time from onset, Mean [SD]	14.4 (18.2)	15.6 (19.0)	11.5 (16.1)	4.1 (-0.5, 8.7)	0.082
Surgery during the last 12 months	n=203	n=139	n=64		
Surgery, n (%)	61 (30.0%)	56 (40.3%)	5 (7.8%)	32.5% (20.7% - 42.0%)	<0.001
Gastroenterologist visits in the past 12 months	n=203	n=139	n=64		
Number of visits, Mean [SD]	4.3 (8.3)	3.5 (3.7)	5.8 (13.7)	-2.3 (-5.8, 1.2)	0.189
Hospitalization in the past 12 months, n (%)	n=203	n=139	n=64		
Hospitalization	36 (17.7%)	24 (17.3%)	12 (18.8%)	-1.5% (-13.4% - 9.5%)	0.844
Comorbidities, n (%)	n=201	n=138	n=63		
One or more	131 (65.2%)	91 (65.9%)	40 (63.5%)	2.4% (-11.5% - 16.8%)	0.752
Disease Activity*, n (%)	n=287	HBI (n=201)	SCCAI (n=86)		
Remission	123 (42.9%)	87 (43.3%)	36 (41.9%)	1.4% (-11.1% - 13.7%)	
Mild	77 (26.8%)	51 (25.4%)	26 (30.2%)	-4.9% (-16.4% - 6.3%)	
Moderate to Severe	87 (30.3%)	63 (30.3%)	24 (27.9%)	3.4% (-8.3% - 14.5%)	0.673
Moderate	83 (28.9%)	60 (29.9%)	23 (26.7%)	3.1% (-8.5% - 14.0%)	
Severe	4 (1.4%)	3 (1.4%)	1 (1.2%)	--	
Ongoing treatment					
Biological agents, n (%)	n=287	n=201	n=86		
TNF inhibitors	191 (66.6%)	145 (72.1%)	46 (53.5%)	18.6% (6.4% - 30.6%)	0.002
Integrin α4 inhibitor	29 (10.1%)	11 (5.5%)	18 (20.9%)	-15.5% [-24.9% - -6.5%)	<0.001
Interleukin-12/23 inhibitor	56 (19.5%)	43 (21.4%)	13 (15.1%)	6.3% (-3.7% - 15.3%)	0.219
JAK inhibitors	11 (3.8%)	2 (1%)	9 (10.5%)	-9.5% (-16.7% - -3.1%)	<0.001
Non-biologic agents, n (%)	n=287	n=201	n=86		
5-ASA	65 (22.6%)	14 (7%)	51 (59.3%)	-52.3% (-62.5% - -40.8%)	<0.001
Corticosteroids	28 (9.7%)	15 (7.5%)	13 (15.1%)	-7.7% (-16.5% - 0.5%)	0.045
Immunosuppressants	56 (19.5%)	36 (17.9%)	20 (23.3%)	-5.3% (-16.0% - 4.7%)	0.295
Antibiotics	11 (3.8%)	8 (4%)	3 (3.5%)	0.5% (-6.1% - 4.8%)	0.842

5-ASA, 5-aminosalicylic acid; BMI, body mass index; CD, Crohn’s disease; JAK, Janus kinase; SD, standard deviation; TNF, tumor necrosis factor; UC, ulcerative colitis.

*SCCAI [remission: <2; mild: 3 to 5; moderate: 6 to 11; severe: >11], HBI [remission: 0-4; mild: 5-7; moderate: 8-16; severe:>16].

^§^Full- or part-time employment or self-employed.

^a^Unadjusted mean difference (95% CI) between groups for continuous variables and difference in percentage points (95% CI) between groups for categorical variables.

^b^Pearson’s χ^2^ test or Mann-Whitney test.

At the time of assessment, 42.9% of patients treated with advanced therapies were in remission, 26.8% had mild disease and 30.3% had moderate-to-severe disease. Overall, 66.6% were treated with TNF inhibitors, 19.5% with IL-12/23 inhibitors, 10.1% with integrin α4 inhibitor, and 3.8% with Janus kinase inhibitors (**Table 1**). A proportion of patients were also receiving non-advanced therapy like 5-ASAs (22.6%), corticosteroids (9.7%), immunosuppressants (19.5%) and antibiotics (3.8%).

Notably, a significantly higher proportion of CD patients had undergone surgery in the preceding 12 months compared to UC patients (40.3% vs 7.8%, p <0.001). In addition, current smoking was more prevalent among CD patients than among UC patients (41.3% vs. 20.9%, p = 0.001) (**Table 1**).

### 3.2 Patient-reported outcomes

The mean SIBDQ score was estimated at 46.0 ± 15.1, with the majority of participants (76.4%) reporting moderately to severely impaired QoL (**Table 2**). No statistically significant differences were observed between the CD and UC cohorts.

**Table 2:** Patient-reported outcomes in the study population.

PROs	Total	CD (n=201)	UC (n=86)	Difference (95% CI)^a^	p-value^b^
Quality of life					
SIBDQ	n=220	n=148	n=72		
Mean [SD]	46.0 (15.1)	45.4 (15.4)	47.4 (14.5)	-2.0 (-6.3-2.2)	0.351
Moderate to severe impact [<60], n (%)	168 (76.4%)	113 (76.3%)	55 (76.4%)	-0.1 (-11.6% - 12.3%)	0.995
Productivity loss					
WPAI					
Absenteeism	n=116	n=77	n=39		
Mean [SD]	12% (24.9%)	11.8% (23.6%)	12.4% (25.1%)	-0.4% (-10% - 10%)	0.911
Moderate to severe impact [≥20%], n (%)	23 (19.8%)	15 (19.5%)	8 (20.5%)	-1.0% (-17.2% - 13.8%)	0.895
Presenteeism	n=110	n=74	n=36		
Mean [SD]	22.9% (26.2%)	22.4% (25.1%)	23.9% (28.5%)	-1.4% (-12.0% - 9.1%)	0.786
Moderate to severe impact [≥20%], n (%)	53 (48.2%)	28 (51.4%)	15 (41.7%)	9.7% (-10.1%, 28.5%)	0.34
Work productivity loss	n=116	n=77	n=39		
Mean [SD]	30.3% (32.7%)	29.4% (31.4%)	32.2% (35.5%)	-2.7% (-15.5% - 10.0%)	0.671
Moderate to severe impact [≥20%], n (%)	60 (51.7%)	42 (54.5%)	18 (46.2%)	8.4% (-10.7% - 16.9)	0.393
Activity impairment	n=212	n=145	n=67		
Mean [SD]	38.4% (32.3%)	37.9% (32.0%)	39.4% (31.2%)	-1.4% (-10.9% - 7.9%)	0.758
Moderate to severe impact [≥20%], n (%)	143 (67.5%)	99 (68.3%)	44 (65.7%)	2.6% (-10.7% - 16.3%)	0.707
Psychological burden					
PHQ-9	n=212	n=145	n=67		
Mean [SD]	10.2 (7.7)	10.1 (7.7)	10.4 (7.9)	-0.3 (-2.5-2)	0.815
Moderate to severe impact [≥10], n (%)	102 (48.1%)	68 (46.9%)	34 (50.7%)	-3.8% (-18.1%, 10.5%)	0.602
Treatment satisfaction	n=271	n=188	n=83		
Yes, n (%) ^¥^	165 (60.9%)	110 (58.5%)	55 (66.3%)	-7.8% (-19.7%, 4.8%)	0.142
Treatment adherence	n=266	n=188	n=83		
Yes, n (%) ^§^	237 (89.1%)	174 (95.1%)	63 (75.9%)	19.2% (9.6%, 29.0%)	<0.001

CD, Crohn’s disease; PHQ-9, Patient Health Questionnaire-9; PROs, Patient-Reported Outcomes; SD, standard deviation; SIBDQ, Short Inflammatory Bowel Disease Questionnaire; UC, ulcerative colitis.

^¥^Responses for “a lot/very much satisfied”.

^§^Responses for “I follow my treatment regularly”.

^a^Unadjusted mean difference (95% CI) between groups for continuous variables and difference in percentage points (95% CI) between groups for categorical variables.

^b^Pearson’s χ2 test or Mann-Whitney test.

Regarding the productivity loss, the mean absenteeism was 12% ± 24.9%, presenteeism 22.9% ± 26.2%, work productivity loss 30.3% ± 32.7%, and activity impairment 38.4% ± 32.3% with comparable values between the two cohorts (**Table 2**). Moderate-to-severe absenteeism, presenteeism, work productivity and activity impairment were reported by 19.8%, 48.2%, 51.7%, 67.5% patients, respectively (**Table 2**).

The mean PHQ-9 score was 10.2 ± 7.7, exceeding the threshold (≥10) for moderate-to-severe depression (**Table 2**). Notably, nearly half of the patients (48.1%) presented with moderate-to-severe depressive symptoms.

Importantly, 39.1% of the participants reported being “not at all,” “little” or only “quite” satisfied with their advanced therapy (**Table 2**, **Supplementary Table 2**). “Increasing fatigue” was the most frequently reported reason for dissatisfaction (20.5%), followed by “recurrent flares” (15.3%) and “frequent bowel movements” (14.9%) (**Supplementary Table 2**). Overall, 10.9% of the population was non-adherent to treatment, with a significantly higher proportion among UC patients compared with CD patients (24.1% vs. 4.9%, p <0.001) (**Table 2**, **Supplementary Table 3**). The most commonly reported reason for non-adherence was patients’ perception that their symptoms were under control (**Supplementary Table 3**).

### 3.3 Univariate and multivariate analysis

Univariate and multivariate logistic regressions were conducted to identify factors associated with moderately to severely impaired QoL [SIBDQ < 60], moderate to severe overall work impairment [WPAI ≥ 20%], moderate to severe depressive symptoms [PHQ-9 ≥ 10], as well as treatment satisfaction and adherence (**Tables 3**–**5**, **Supplementary Tables 4**, **5**).

**Table 3:** Factors associated with moderately to severely [SIBDQ <60] impaired QoL: univariate and multivariate logistic regressions analyses.

SIBDQ	Univariate analysis		Multivariate analysis	
	OR [95% CI]	p-value	OR [95% CI]	p-value
Gender				
Male	Ref		Ref	
Female	3.68 [1.91-7.08]	<0.001	2.218 (1.140-4.314)	0.049
Age				
<50 years	Ref			
50 years or more	1.131 (0.554 – 2.309)	0.735		
Employment status				
In paid employment	Ref		Ref	
Other	3.286 (1.596 – 6.768)	0.001	2.442 (1.168-5.103)	0.046
BMI				
Underweight and normal	Ref			
Overweight and obese	1.143 (0.612 -2.135)	0.675		
Smoking status				
Never smoker	Ref		Ref	
Former smoker	1.174 (0.559 - 2.465)	0.671	0.881 (0.389 - 1.998)	0.8
Current smoker	2.037 (0.939 - 4.421)	0.072	1.304 (0.564 - 3.018)	0.603
Disease activity				
Inactive*	Ref		Ref	
Active**	15.171 (6.405 - 35.934)	<0.001	10.751 (4.976-23.231)	<0.001
Age at diagnosis				
0–30 years	Ref		Ref	
>30 years	1.827 (0.950 - 3.514)	0.071	0.907 (0.420 – 1.956)	0.835
Disease duration				
<10 years	Ref			
>10years	0.852 (0.456 – 1.592)	0.615		
Surgery				
No	Ref			
Yes	1.254 (0.610 – 2.576)	0.538		
Disease				
CD	Ref			
UC	1.002 (0.516 -1.945)	0.995		
Comorbidities				
None	Ref		Ref	
One or more	3.111 (1.601-6.046)	<0.001	1.664 (0.846 – 3.275)	0.216

CLI, confidence interval; BMI, body mass index; N/A, not applicable; OR, odds ratio; ref, reference value; SIBDQ, Short Inflammatory Bowel Disease Questionnaire.

*Patients in remission.

**Patients with mild, moderate or severe disease activity.

**Table 4:** Factors associated with moderate to severe overall work impairment (WPAI ≥20%): univariate and multivariate logistic regressions analyses.

WPAI	Univariate analysis		Multivariate analysis	
	OR [95% CI]	p-value	OR [95% CI]	p-value
Gender				
Male	Ref		Ref	
Female	2.52 (1.19 – 5.33)	0.016	2.190 (1.045 – 4.588)	0.081
Age				
<50 years	Ref			
50 years or more	1.01 (0.42 – 2.46)	0.975		
Employment status				
Paid employment	Ref			
Other	3.00 (0.30 – 29.76)	0.348		
BMI				
Underweight and normal	Ref			
Overweight and obese	1.13 (0.54 – 2.36)	0.746		
Smoking status				
Never	Ref		Ref	
Former smoker	0.956 (0.385 – 2.373)	0.922	0.907 (0.368 – 2.233)	0.858
Current smoker	2.248 (0.924 – 5.472)	0.074	1.90 (0.764 – 4.725)	0.247
Disease activity				
Inactive*	Ref		Ref	
Active**	9.096 (3.911 – 21.153)	<0.001	8.700 (4.028 – 18.791)	<0.001
Age at diagnosis				
0–30 years	Ref		Ref	
>30 years	2.455 (1.114 – 5.410)	0.026	1.050 (0.455 – 2.425)	0.923
Disease duration				
<10 years	Ref		Ref	
>10 years	0.480 (0.227 – 1.017)	0.055	0.577 (0.269 – 1.240)	0.237
Surgery				
No	Ref			
Yes	1.746 (0.764 – 3.990)	0.186		
Disease				
CD	Ref			
UC	0.714 (0.330 -1.547)	0.384		
Comorbidities				
No	Ref			
One or more	1.327 (0.622 – 2.830)	0.464		

BMI, body mass index; CI, confidence interval; OR, odds ratio; ref, reference value; WPAI, Work Productivity and Activity Impairment.

*Patients in remission.

**Patients with mild, moderate or severe disease activity.

**Table 5:** Factors associated with PHQ-9: univariate and multivariate logistic regressions analyses.

PHQ-9	Univariate analysis		Multivariate analysis	
	OR [95% CI]	p-value	OR [95% CI]	p-value
Gender				
Male	Ref		Ref	
Female	4.02 (1.94 -8.34)	<0.001	2.204 (1.256 – 3.868)	0.021
Age				
<50 years	Ref			
50 years or more	0.878 (0.480 – 1.603)	0.671		
Employment status				
In paid employment	Ref		Ref	
Other	1.781 (1.023-3.101)	0.041	1.210 (0.695 – 2.106)	0.572
BMI				
Underweight and normal	Ref			
Overweight and obese	1.139 (0.663 – 1.960)	0.637		
Smoking status				
Never	Ref		Ref	
Former smoker	1.393 (0.706 – 2.749)	0.339	1.661 (0.832 – 3.318)	0.228
Current smoker	1.832 (0.953 – 3.519)	0.069	1.699 (0.882 – 3.272)	0.184
Disease activity				
Inactive*	Ref		Ref	
Active**	6.750 (3.640 – 12.516)	<0.001	6.343 (3.595 – 11.193)	<0.001
Age at diagnosis				
0–30 years	Ref			
>30 years	1.098 (0.637 – 1.893)	0.735		
Disease duration				
<10 years	Ref			
>10 years	0.899 (0.524 - 1.542)	0.699		
Surgery				
No	Ref			
Yes	0.794 (0.435 – 1.451)	0.454		
Disease				
CD	Ref			
UC	1.167 (0.654 – 2.083)	0.602		
Comorbidities				
No	Ref		Ref	
One or more	3.125 (1.686 – 5.794)	<0.001	1.871 (1.032 – 3.391)	0.083

BMI, body mass index; CI, confidence interval; OR, odds ratio; ref, reference value; PHQ-9, Patient Health Questionnaire.

*Patients in remission.

**Patients with mild, moderate or severe disease activity.

In multivariate analysis, the risk of moderately to severely impaired QoL was significantly higher among women (OR: 2.21, [95%CI: 1.14-4.31], p= 0.049) and among patients not in paid employment (2.44, [1.16–5.10], p= 0.046). Patients with active disease had nearly eleven times higher odds of impaired QoL (10.75, [4.97–23.23], p < 0.001) and almost nine times higher odds of moderate to severe overall work impairment (8.7, [4.02–18.79], p < 0.001) compared to those in remission (**Tables 3**, **4**). Multivariate analysis further demonstrated that both sex and disease severity were independently associated with depression. Specifically, female sex (2.2, [1.25–3.86], p = 0.02) and active disease (6.34, [3.59–11.19], p < 0.001) were identified as factors significantly associated with higher odds of moderate to severe depressive symptoms (**Table 5**).

Regarding treatment satisfaction, patients with active disease were significantly less likely to report being satisfied with their treatment compared with those in remission (0.28, [0.16–0.49], p < 0.001) (**Supplementary Table 4**). Notably, multivariate analysis also indicated that UC patients had significantly lower odds of adhering to treatment compared to CD patients (0.24, [0.10–0.54], p = 0.004) (**Supplementary Table 5**).

### 3.4 Correlations between PROs and disease activity

**Figure 1:**
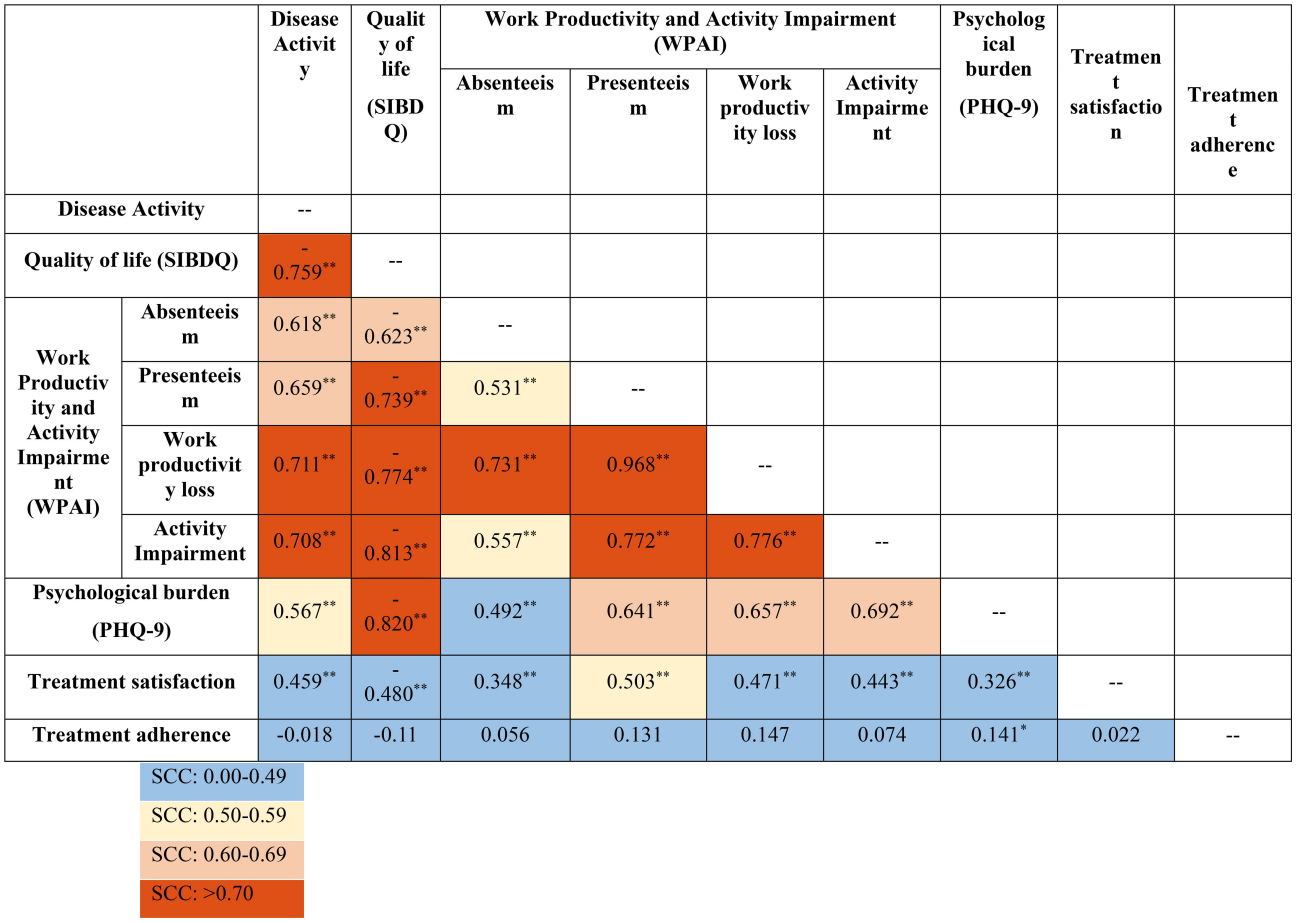
Spearman’s correlation coefficient (SCC) for patient-reported outcomes and disease activity for all patients receiving biologic therapy. PHQ-9, Patient Health Questionnaire-9; SIBDQ, Short Inflammatory Bowel Disease Questionnaire; WPAI, Work Productivity and Activity Impairment Questionnaire. **Correlation is significant at the 0.01 level (2-tailed). *Correlation is significant at the 0.05 level (2-tailed).

All the SCCs demonstrated statistically significant (P<0.01) associations between PROs and disease activity (**Figure 1**).

QoL was strongly and inversely associated with psychological burden (SCC: -0.820) and with activity impairment (-0.813), representing two of the strongest correlations observed in the analysis. QoL also demonstrated consistent negative correlations with all WPAI domains, including work productivity loss (-0.774), presenteeism (-0.739) and absenteeism (-0.623) (**Figure 1**).

Disease activity showed a strong negative correlation with QoL (-0.759) (**Figure 1**). In parallel, disease activity was positively correlated with all WPAI domains, including work productivity loss (0.711), activity impairment (0.708), presenteeism (0.659), and absenteeism (0.618) (**Figure 1**). A moderate positive association was also observed between disease activity and psychological burden (0.567).

Within the WPAI domains, the strongest correlation was observed between presenteeism and work productivity loss (0.968) (**Figure 1**). Work productivity loss was also strongly associated with absenteeism (0.731) and activity impairment (0.776), while a strong correlation was additionally observed between activity impairment and presenteeism (0.772).

Psychological burden demonstrated consistent positive correlations with all WPAI domains, with the strongest associations observed for activity impairment (0.692), work productivity loss (0.657), and presenteeism (0.641) (**Figure 1**).

Correlations between treatment satisfaction and other outcomes were generally moderate to weak. Moderate correlations were observed with presenteeism (0.503), work productivity loss (0.471), and activity impairment (0.443). Treatment satisfaction was also moderately correlated with disease activity (0.459) and inversely correlated with QoL (-0.480). Weaker correlations were identified with absenteeism (0.348) and psychological burden (0.326). No significant correlations were observed between treatment adherence and other PROs or disease activity, except for a weak association with psychological burden (0.141).

## 4 Discussion

Despite major advances in IBD management, particularly the broad use of biologic therapies, a substantial disease burden persists that extends beyond gastrointestinal symptoms and negatively affects QoL, work productivity and mental well-being. In this context, PROs have become integral to contemporary IBD research and clinical practice, serving as key endpoints in clinical trials and essential tools for capturing the broader patient-perceived impact of disease in real-world settings (39, 40). Accordingly, the present study aimed to assess the multidimensional impact of IBD among patients receiving advanced therapies and to identify unmet medical needs in a Greek real-world setting, where available evidence remains limited.

At the time of assessment, all participants were receiving advanced therapies, with TNF inhibitors being the most commonly used treatment (66.6%). Despite biologic therapy, disease control remained suboptimal, with only 42.9% of patients in remission, while 26.8% had mild disease and 30.3% had moderate-to-severe disease activity. These findings align with real-world and meta-analytic evidence indicating that remission is achieved in approximately 40-60% of patients receiving biologics, leaving a considerable proportion with ongoing disease activity (41). This persistent clinical burden was further reflected in impaired PROs across multiple domains.

The mean SIBDQ score of 46.0 indicated impaired QoL among both CD and UC patients receiving advanced therapies, with more than three-quarters reporting moderate-to-severe impairment. These findings are consistent with previous data from Greece showing that individuals with IBD experience substantially lower health-related QoL compared with the general population, even while receiving treatment (42). Multivariate analysis further demonstrated an inverse association between disease activity and QoL, in line with previous evidence (42, 43). Importantly, patients not engaged in paid employment and women were more likely to report moderately to severely impaired QoL, consistent with existing literature (44).

A substantial work-related burden was also observed. Moderate-to-severe absenteeism, presenteeism, work productivity and activity impairment were reported by 19.8%, 48.2%, 51.7%, 67.5% patients, respectively. Considering that 54.9% of participants were in paid employment, these findings highlight the ongoing socioeconomic impact of IBD, even in the era of advanced targeted therapies. Our results are consistent with a systematic review and meta-analysis showing that work productivity impairment remains a significant and ongoing challenge in IBD, across diverse clinical populations (19). Likewise, a Greek survey demonstrated that IBD interfered with working capacity in 40% of patients, while 57% required time off work (45). Multivariate analysis further demonstrated that active disease was independently associated with greater work impairment, consistent with prior evidence identifying disease activity as a key factor associated with poor work outcomes in patients with IBD (19, 42).

Psychological distress also represented a major component of disease burden. Based on the PHQ-9 scores, nearly half of patients were associated with moderate-to-severe depressive symptoms. These findings are consistent with previous evidence demonstrating high rates of depression and anxiety among individuals with IBD, particularly during periods of active disease (46). A Greek cross-sectional study similarly highlighted the broad psychosocial impact of IBD and emphasized the importance of multidisciplinary care, including mental health support (47). In line with previous studies, multivariate analysis showed that female sex and active disease were independently associated with a higher likelihood of depressive symptoms in IBD (43, 48).

Treatment dissatisfaction was reported by 39.1% of participants, with worsening fatigue identified as the main reason. This finding is consistent with a recent meta-analysis showing modest fatigue reduction while on biological treatment (49), indicating that fatigue remains a significant challenge in IBD despite the availability of advanced therapies (50). Furthermore, multivariate analysis showed that patients with active disease were less likely to report treatment satisfaction, in line with previous evidence linking persistent symptoms, such as pain, discomfort, fatigue and low energy, to lower satisfaction levels (8).

Overall non-adherence in our cohort was relatively low (10.9%), consistent with evidence suggesting that patients receiving advanced therapies demonstrate higher adherence rates (51). However, non-adherence was markedly higher among patients with UC compared with those with CD (24.1% vs. 4.9%), which was also confirmed by the multivariate analysis. A recent meta-analysis including over 40,000 patients with IBD reported higher adherence to biologic therapies among patients with CD compared to UC, supporting the observed difference in our cohort (52).

The observed correlations between PROs and disease activity highlight the following interconnections: (i) poorer QoL was associated with greater work productivity loss and activity impairment, and higher levels of depression; (ii) higher disease activity was associated with decreased QoL, lower work productivity, and activity impairment, and increased levels of depression; (iii) patients experiencing greater productivity loss and activity impairment were more likely to report depressive symptoms. These findings are consistent with previous studies demonstrating strong relationships between disease activity, impaired QoL, work productivity loss, and psychological distress in IBD populations (42, 43, 53, 54).

Multimorbidity also contributed to the overall disease burden. In our cohort, 41.3% of patients reported at least two comorbidities, with arthritis being the most common. This finding reflects the systemic inflammatory nature of IBD and the significant impact of extraintestinal manifestations on patient outcomes (55, 56).

Overall, no meaningful differences were identified between UC and CD patients receiving advanced therapies across key patient-reported outcomes, including QoL, work-related burden, psychological distress and treatment dissatisfaction. These findings suggest that the broader lived experience of patients undergoing advanced therapies is largely comparable between the two conditions, an observation consistent with previously published evidence (14, 57, 58).

This study has several strengths. It comprehensively assessed multiple PRO domains among patients receiving advanced therapies in a Greek real-world setting, providing insights into the persistent burden of IBD beyond clinical disease activity. The use of both validated and study-specific tools, allowed for a broad and nuanced assessment of QoL, work productivity, psychological well-being, treatment satisfaction, and adherence. Overall, this study underscores the importance of systematically incorporating PRO assessment into routine clinical practice and therapeutic decision-making to support improved health outcomes and more patient-centered care.

Several limitations should also be acknowledged. The cross-sectional design of the study precludes causal inference; therefore, the observed relationships between disease activity and PROs should be interpreted as associations rather than evidence of causal effects, while the possibility of reverse causation or bidirectional relationships cannot be excluded. In addition, the recruitment of participants through the HELLESCC patient association may introduce selection bias. Nevertheless, it should be stressed that HELLESCC represents the official national patient association in Greece and is a member of the European Federation of Crohn’s & Ulcerative Colitis Associations (EFCCA), supporting the broader relevance of the study cohort. In addition, reliance on self-reported information may be subject to recall or reporting bias, while objective markers of disease activity were not included. An additional limitation of the study is that detailed treatment history, including previous biologic exposure, number of prior treatment lines, and treatment failures, was not captured in the study questionnaire. Consequently, the potential influence of prior treatment exposure and treatment refractoriness on disease burden and PROs could not be explored. Finally, treatment satisfaction and adherence were assessed using study-specific questionnaires, which may affect measurement reliability; yet there are no “one-way” tools at present (36, 37, 59) and, despite their limitations, these effectively captured patient experiences.

## 5 Conclusion

In conclusion, this real-world, patient-reported survey demonstrates that a substantial and multidimensional burden persists among Greek patients with UC and CD despite treatment with advanced therapies. Impaired QoL, marked work and activity limitations, and a high prevalence of depressive symptoms highlight the profound impact of IBD beyond clinical disease activity. At the same time, the considerable proportion of patients reporting dissatisfaction with treatment points to a persistent gap between therapeutic targets and outcomes that matter most to patients.

Taken together, these findings reinforce the need to move beyond a solely disease-activity–focused model of care toward a more integrated, patient-centered approach that systematically incorporates patient-reported outcomes into routine clinical practice. Addressing the complex and interrelated physical, psychological, and functional consequences of IBD will require coordinated multidisciplinary management and stronger patient–physician partnerships in therapeutic decision-making. Importantly, the results also have broader health system implications, underscoring the need for policies that recognize the full societal and economic burden of IBD, support access to comprehensive care, and embed patient perspectives in clinical pathways and resource planning. Such efforts are essential to ensure that advances in therapy translate into meaningful improvements in long-term outcomes and QoL for individuals living with IBD in Greece.

## Data Availability

The original contributions presented in the study are included in the article/**Supplementary Material**. Further inquiries can be directed to the corresponding author.

## References

[1] Bisgaard TH Allin KH Keefer L Ananthakrishnan AN Jess T . Depression and anxiety in inflammatory bowel disease: epidemiology, mechanisms and treatment. Nat Rev Gastroenterol Hepatol. (2022) 19:717–26. doi: 10.1038/s41575-022-00634-6 35732730

[2] Gajendran M Loganathan P Jimenez G Catinella AP Ng N Umapathy C . A comprehensive review and update on ulcerative colitis. Disease-a-Month DM. (2019) 65:100851. doi: 10.1016/j.disamonth.2019.02.004 30837080

[3] Cho CW You MW Oh CH Lee CK Moon SK . Long-term disease course of Crohn's disease: changes in disease location, phenotype, activities, and predictive factors. Gut Liver. (2022) 16:157–70. doi: 10.5009/gnl210118 34456186 PMC8924800

[4] Khan S Sebastian SA Parmar MP Ghadge N Padda I Keshta AS . Factors influencing the quality of life in inflammatory bowel disease: a comprehensive review. Disease-a-Month. (2024) 70:101672. doi: 10.1016/j.disamonth.2023.101672 38143196

[5] Fanizzi F Peyrin-Biroulet L Danese S D'Amico F . Targeting the unmet needs in IBD: emerging therapies beyond biologics and small molecules. Curr Opin Pharmacol. (2025) 85:102577. doi: 10.1016/j.coph.2025.102577 41124980

[6] Chen C Hartzema AG Xiao H Wei YJ Chaudhry N Ewelukwa O . Real-world pattern of biologic use in patients with inflammatory bowel disease: treatment persistence, switching, and importance of concurrent immunosuppressive therapy. Inflammatory Bowel Dis. (2019) 25:1417–27. doi: 10.1093/ibd/izz001 30839057

[7] Afzali A Lukanova R Hennessy F Kakehi S Knight H Milligan G . Unmet needs in real-world advanced therapy-naïve and -experienced patients with moderately to severely active ulcerative colitis in the United States. Adv Ther. (2023) 40:4321–38. doi: 10.1007/s12325-023-02605-y 37458875 PMC10499754

[8] Yan X Qiao Y Tong J Mao R Liang J Lv C . Assessment of patient-centered outcomes (PROs) in inflammatory bowel disease (IBD): a multicenter survey preceding a cross-disciplinary (functional) consensus. Health Qual Life Outcomes. (2020) 18:241. doi: 10.1186/s12955-020-01489-8 32690091 PMC7372780

[9] Horrigan JM Louis E Spinelli A Travis S Moum B Salwen-Deremer J . The real-world global use of patient-reported outcomes for the care of patients with inflammatory bowel disease. Crohn's Colitis 360. (2023) 5:otad006. doi: 10.1093/crocol/otad006 36937140 PMC10022710

[10] Tran F Schirmer JH Ratjen I Lieb W Helliwell P Burisch J . Patient reported outcomes in chronic inflammatory diseases: current state, limitations and perspectives. Front Immunol. (2021) 12:614653. doi: 10.3389/fimmu.2021.614653 33815372 PMC8012677

[11] Williet N Sandborn WJ Peyrin–Biroulet L . Patient-reported outcomes as primary end points in clinical trials of inflammatory bowel disease. Clin Gastroenterol Hepatol. (2014) 12:1246–1256.e6. doi: 10.1016/j.cgh.2014.02.016 24534550

[12] Chen XL Zhong LH Wen Y Liu TW Li XY Hou ZK . Inflammatory bowel disease-specific health-related quality of life instruments: a systematic review of measurement properties. Health Qual Life Outcomes. (2017) 15:177. doi: 10.1186/s12955-017-0753-2 28915891 PMC5603012

[13] Nazarian A Bishay K Gholami R Scaffidi MA Khan R Cohen-Lyons D . Factors associated with poor quality of life in a Canadian cohort of patients with inflammatory bowel disease: a cross-sectional study. J Can Assoc Gastroenterol. (2021) 4:91–6. doi: 10.1093/jcag/gwaa014 33855267 PMC8023811

[14] Christiansen LK Lo B Bendtsen F Vind I Vester-Andersen MK Burisch J . Health-related quality of life in inflammatory bowel disease in a Danish population-based inception cohort. United Eur Gastroenterol J. (2019) 7:942–54. doi: 10.1177/2050640619852532 31428419 PMC6683643

[15] Williet N Sarter H Gower-Rousseau C Adrianjafy C Olympie A Buisson A . Patient-reported outcomes in a French nationwide survey of inflammatory bowel disease patients. J Crohn's Colitis. (2017) 11:165–74. doi: 10.1093/ecco-jcc/jjw145 27516406

[16] Irvine EJ Zhou Q Thompson AK CCRPT Investigators Canadian Crohn's Relapse Prevention Trial . The Short Inflammatory Bowel Disease Questionnaire: a quality of life instrument for community physicians managing inflammatory bowel disease. Am J Gastroenterol. (1996) 91:1571–8. doi: 10.1097/00004836-199201000-00005 8759664

[17] Reilly MC Zbrozek AS Dukes EM . The validity and reproducibility of a work productivity and activity impairment instrument. PharmacoEconomics. (1993) 4:353–65. doi: 10.2165/00019053-199304050-00006 10146874

[18] Cross RK Sauk JS Zhuo J Harrison RW Kerti SJ Emeanuru K . Poor patient-reported outcomes and impaired work productivity in patients with inflammatory bowel disease in remission. Gastro Hep Adv. (2022) 1:927–35. doi: 10.1016/j.gastha.2022.07.003 39131245 PMC11307635

[19] Youssef M Hossein-Javaheri N Hoxha T Mallouk C Tandon P . Work productivity impairment in persons with inflammatory bowel diseases: a systematic review and meta-analysis. J Crohn's Colitis. (2024) 18:1486–504. doi: 10.1093/ecco-jcc/jjae057 38647194 PMC11369077

[20] Byrne G Rosenfeld G Leung Y Qian H Raudzus J Nunez C . Prevalence of anxiety and depression in patients with inflammatory bowel disease. Can J Gastroenterol Hepatol. (2017) 2017:6496727. doi: 10.1155/2017/6496727 29181373 PMC5664260

[21] Kroenke K Spitzer RL Williams JB . The PHQ-9: validity of a brief depression severity measure. J Gen Internal Med. (2001) 16:606–13. doi: 10.1046/j.1525-1497.2001.016009606.x 11556941 PMC1495268

[22] Marín-Jiménez I Aguirregabiria I Díaz-Cerezo S Moyano S Gabilondo H Knight H . Unmet needs in adult patients with ulcerative colitis in Spain: a real-world Adelphi Disease Specific Programme study. (2025) 18:17562848251325190. doi: 10.1177/17562848251325190 PMC11956514 40166589

[23] Schönborn C Levy M De Jaeger M Van Goethem R Leonard U Claerman R . Unmet health-related needs in patients with Crohn’s disease in Belgium: a mixed-methods study. Arch Public Health. (2025) 83:151. doi: 10.1186/s13690-025-01632-1 40514703 PMC12164149

[24] Kershaw J Sanon M Kachroo S Barlow S Naessens D Willey CJ . Real-world impact of uncontrolled symptoms and suboptimal treatment response in patients with Crohn’s disease in the United States and Europe. Crohn's Colitis 360. (2024) 7(1):otae074. doi: 10.1093/crocol/otae074 39834357 PMC11744185

[25] Gourzoulidis G Vakouftsi VR Mavridoglou G Psarra M Tzanetakos C . Disease burden and unmet medical needs in patients with ulcerative colitis in Greece: a cross-sectional patient survey. Med Sci (Basel Switzerland). (2025) 13(3):117. doi: 10.3390/medsci13030117 40843739 PMC12371981

[26] Tzanetakos C Vakouftsi VR Mavridoglou G Psarra M Gourzoulidis G . Disease burden and unmet medical need in patients with Crohn's disease in Greece: a cross-sectional patient survey. Ann Gastroenterol. (2025) 38:629–40. doi: 10.20524/aog.2025.1013 41586393 PMC12829537

[27] Viazis N Stefanidou A Mantzaris GJ . The ulcerative colitis narrative Greece survey: patients' and physicians' perspective on quality of life and disease management. Ann Gastroenterol. (2022) 35:267–74. doi: 10.20524/aog.2022.0708 35599924 PMC9062848

[28] Argyriou K Kapsoritakis A Oikonomou K Manolakis A Tsakiridou E Potamianos S . Disability in patients with inflammatory bowel disease: correlations with quality of life and patient's characteristics. Can J Gastroenterol Hepatol. (2017) 2017:6138105. doi: 10.1155/2017/6138105 28634576 PMC5467285

[29] Walmsley RS Ayres RC Pounder RE Allan RN . A simple clinical colitis activity index. Gut. (1998) 43:29–32. doi: 10.1136/gut.43.1.29 9771402 PMC1727189

[30] Walsh AJ Ghosh A Brain AO Buchel O Burger D Thomas S . Comparing disease activity indices in ulcerative colitis. J Crohn's Colitis. (2014) 8:318–25. doi: 10.1016/j.crohns.2013.09.010 24120021

[31] Jowett SL Seal CJ Phillips E Gregory W Barton JR Welfare MR . Defining relapse of ulcerative colitis using a symptom-based activity index. Scandinavian J Gastroenterol. (2003) 38:164–71. doi: 10.1080/00365520310000654 12678333

[32] Best WR . Predicting the Crohn's disease activity index from the Harvey-Bradshaw Index. Inflammatory Bowel Dis. (2006) 12:304–10. doi: 10.1097/01.MIB.0000215091.77492.2a 16633052

[33] Biedermann L Fournier N Misselwitz B Frei P Zeitz J Manser CN . High rates of smoking especially in female Crohn’s disease patients and low use of supportive measures to achieve smoking cessation—data from the Swiss IBD Cohort Study. J Crohn's Colitis. (2015) 9:819–29. doi: 10.1093/ecco-jcc/jjv113 26116554

[34] Keller R Mazurak N Fantasia L Fusco S Malek NP Wehkamp J . Quality of life in inflammatory bowel diseases: it is not all about the bowel. Intestinal Res. (2021) 19:45–52. doi: 10.5217/ir.2019.00135 32093437 PMC7873402

[35] Hua X Lopes EW Burke KE Ananthakrishnan AN Richter JM Lo CH . Smoking behaviour changes after diagnosis of inflammatory bowel disease and risk of all-cause mortality. J Crohn's Colitis. (2022) 16:1030–8. doi: 10.1093/ecco-jcc/jjac015 35102373 PMC9351977

[36] King K Norton C Chalder T Czuber-Dochan W . N17 exploration of medication non-adherence in inflammatory bowel disease patients: a systematic review. J Crohn's Colitis. (2024) 18:i2207–7. doi: 10.1093/ecco-jcc/jjad212.1389

[37] de Castro ML Sanromán L Martín A Figueira M Martínez N Hernández V . Assessing medication adherence in inflammatory bowel diseases. A comparison between a self-administered scale and a pharmacy refill index. Rev Espanola Enfermedades Digestivas. (2017) 109:542–51. doi: 10.17235/reed.2017.5137/2017 28679280

[38] du Prel JB Hommel G Röhrig B Blettner M . Confidence interval or p-value?: part 4 of a series on evaluation of scientific publications. Deutsches Arzteblatt Int. (2009) 106:335–9. doi: 10.3238/arztebl.2009.0335 19547734 PMC2689604

[39] Sandborn WJ Vermeire S Peyrin-Biroulet L Dubinsky MC Panes J Yarur A . Etrasimod as induction and maintenance therapy for ulcerative colitis (ELEVATE): two randomised, double-blind, placebo-controlled, phase 3 studies. Lancet. (2023) 401:1159–71. doi: 10.1016/S0140-6736(23)00061-2 36871574

[40] Danese S Vermeire S Zhou W Pangan AL Siffledeen J Greenbloom S . Upadacitinib as induction and maintenance therapy for moderately to severely active ulcerative colitis: results from three phase 3, multicentre, double-blind, randomised trials. Lancet. (2022) 399:2113–28. doi: 10.1016/S0140-6736(22)00581-5 35644166

[41] Shah-Riar P Tamanna N Kulsum U Gould A Zamir A . Long-term outcomes of biologic therapy in IBD: a meta-analysis of real-world and randomized data. Gastroenterology. (2026) 170:S76–7. doi: 10.1053/j.gastro.2025.10.128

[42] Kafalis N Kogias D Papadopoulos VP Moschos I Skendros P Ritis K . Impact of inflammatory bowel disease on quality of life and work productivity in patients under treatment. Cureus. (2025) 17:e97297. doi: 10.7759/cureus.97297 41426881 PMC12717476

[43] Swaminathan A Fan D Borichevsky GM Mules TC Hirschfeld E Frampton CM . The disease severity index for inflammatory bowel disease is associated with psychological symptoms and quality of life, and predicts a more complicated disease course. Alimentary Pharmacol Ther. (2022) 56:664–74. doi: 10.1111/apt.17058 35633043 PMC9545845

[44] Roukas C Miller L Cléirigh Büttner F Hamborg T Stagg I Hart A . Impact of pain, fatigue and bowel incontinence on the quality of life of people living with inflammatory bowel disease: a UK cross-sectional survey. (2025) 13:364–75. doi: 10.1002/ueg2.12668 PMC7616848 39425758

[45] Viazis N Mantzaris G Karmiris K Polymeros D Kouklakis G Maris T . Inflammatory bowel disease: Greek patients' perspective on quality of life, information on the disease, work productivity and family support. Ann Gastroenterol. (2013) 26:52–8. PMC3959518 24714294

[46] Bartocci B Dal Buono A Gabbiadini R Busacca A Quadarella A Repici A . Mental illnesses in inflammatory bowel diseases: mens sana in corpore sano. Med (Kaunas Lithuania). (2023) 59(4):682. doi: 10.3390/medicina59040682 37109640 PMC10145199

[47] Mitropoulou MA Fradelos EC Lee KY Malli F Tsaras K Christodoulou NG . Quality of life in patients with inflammatory bowel disease: importance of psychological symptoms. Cureus. (2022) 14:e28502. doi: 10.7759/cureus.28502 36185946 PMC9514670

[48] Fracas E Costantino A Vecchi M Buoli M . Depressive and anxiety disorders in patients with inflammatory bowel diseases: are there any gender differences? Int J Environ Res Public Health. (2023) 20(13):6255. doi: 10.3390/ijerph20136255 37444101 PMC10340762

[49] Skjellerudsveen BM Skoie IM Dalen I Grimstad T Omdal R . The effect of biological treatment on fatigue in inflammatory bowel disease: a systematic review and meta-analysis. Drugs. (2023) 83:909–21. doi: 10.1007/s40265-023-01888-3 37219801 PMC10284989

[50] Włodarczyk M Makaro A Prusisz M Włodarczyk J Nowocień M Maryńczak K . The role of chronic fatigue in patients with Crohn's disease. Life (Basel Switzerland). (2023) 13(8):1692. doi: 10.3390/life13081692 37629549 PMC10455565

[51] Stroie TG Diaconescu LV Preda C Diculescu M Chirea TM Istratescu D . Treatment adherence in inflammatory bowel disease: The role of demographic, clinical, and psychosocial factors. (2025) 61:1512. doi: 10.3390/medicina61091512 PMC12472098 41010903

[52] Pan W Tian W Li S Zhao Y Liu Y He Y . Adherence to biologics in patients with inflammatory bowel disease: a systematic review and meta-analysis. Int J Clin Pharm. (2026) 48:51–66. doi: 10.1007/s11096-025-02061-4 41369787

[53] Aniwan S Bruining DH Park SH Al-Bawardy B Kane SV Coelho Prabhu N . The combination of patient-reported clinical symptoms and an endoscopic score correlates well with health-related quality of life in patients with ulcerative colitis. J Clin Med. (2019) 8(8):1171. doi: 10.3390/jcm8081171 31387259 PMC6723355

[54] Jackson BD Con D Gorelik A Liew D Knowles S De Cruz P . Examination of the relationship between disease activity and patient-reported outcome measures in an inflammatory bowel disease cohort. Internal Med J. (2018) 48:1234–41. doi: 10.1111/imj.13937 29663629

[55] Mosli MH Alsahafi M Alsanea MN Alhasani F Ahmed M Saadah O . Multimorbidity among inflammatory bowel disease patients in a tertiary care center: a retrospective study. BMC Gastroenterol. (2022) 22:487. doi: 10.1186/s12876-022-02578-2 36435785 PMC9701410

[56] Arvikar SL Fisher MC . Inflammatory bowel disease associated arthropathy. Curr Rev Musculoskeletal Med. (2011) 4:123–31. doi: 10.1007/s12178-011-9085-8 21710141 PMC3261248

[57] Ding Z Muser E Izanec J Lukanova R Kershaw J Roughley A . Work-related productivity loss and associated indirect costs in patients with Crohn's disease or ulcerative colitis in the United States. Crohn's Colitis 360. (2022) 4:otac023. doi: 10.1093/crocol/otac023 36777416 PMC9802455

[58] Parra RS Chebli JMF Amarante H Flores C Parente JML Ramos O . Quality of life, work productivity impairment and healthcare resources in inflammatory bowel diseases in Brazil. World J Gastroenterol. (2019) 25:5862–82. doi: 10.3748/wjg.v25.i38.5862 31636478 PMC6801193

[59] Hubscher E Vurgun N . PCR152 underutilization of a validated treatment satisfaction instrument in inflammatory bowel disease. Value Health. (2022) 25:S420. doi: 10.1016/j.jval.2022.09.2087 38826717

