# Supplementary material for: Persistent disease burden despite advanced therapies in inflammatory bowel disease: a real-world patient-reported survey from Greece
Source: Front Immunol. 2026 Jul 10;17:1866461. doi: 10.3389/fimmu.2026.1866461 (PMC13396155; doi:10.3389/fimmu.2026.1866461)
Supplement: Supplementary file 1 [file Supplementaryfile1.docx]

**Supplementary material**

**Supplementary Figure S1**. Flow diagram of patient selection and inclusion in the study.


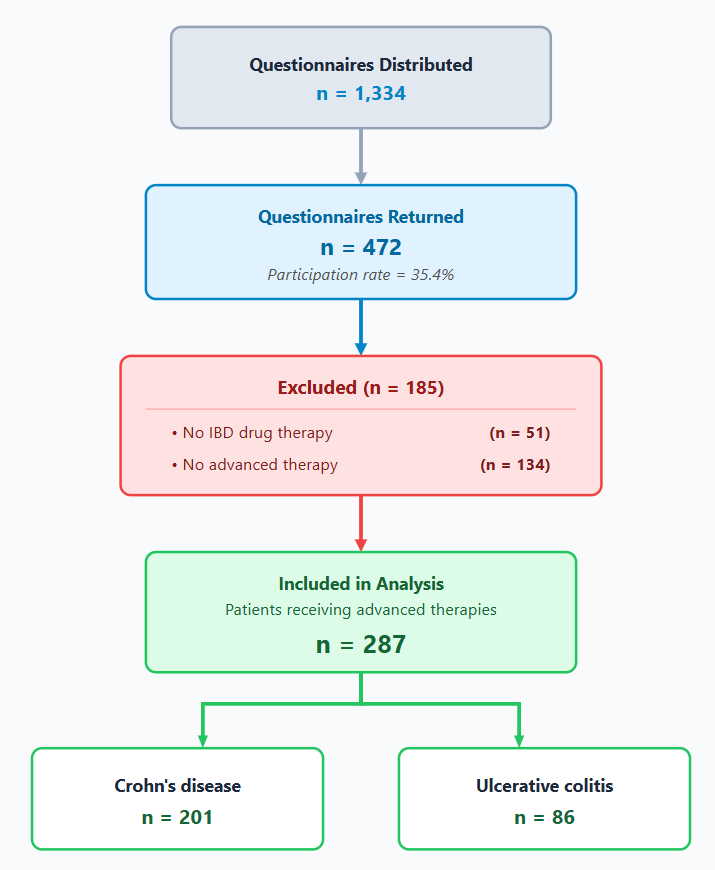


Abbreviations: IBD: Inflammatory Bowel Disease.

**Supplementary Table S1.** Comorbidities Stratified by Disease Type

| **Comorbidities** | **Total**  **(N=** **131)** | **CD**  **(Ν=** **91)** | **UC**  **(Ν=40)** |
| --- | --- | --- | --- |
| COPD | 4 (3,1%) | 3 (3,3%) | 1 (2,5%) |
| Arthritis | 51 (38,9%) | 36 (39,6%) | 15 (37,5%) |
| Iron deficiency anaemia | 30 (22,9%) | 22 (24,2%) | 8 (20%) |
| Hypothyroidism | 22 (16,8%) | 16 (17,6%) | 6 (15%) |
| Skin allergies or other skin conditions | 37 (28,2%) | 29 (31,9%) | 8 (20%) |
| Cancer | 2 (1,5%) | 2 (2,2%) | 0 (0%) |
| Depression | 38 (29%) | 25 (27,5%) | 13 (32,5%) |
| Diabetes | 16 (12,2%) | 11 (12,1%) | 5 (12,5%) |
| Hypertension | 20 (15,3%) | 13 (14,3%) | 7 (17,5%) |
| Cardiovascular disease | 6 (4,6%) | 2 (2,2%) | 4 (10%) |
| Coronary artery disease | 3 (2,3%) | 0 (0%) | 3 (7,5%) |
| Osteoporosis | 15 (11,5%) | 8 (8,8%) | 7 (17,5%) |
| Migraine or severe headache | 14 (10,7%) | 8 (8,8%) | 6 (15%) |
| Other | 36 (27,5%) | 24 (26,4%) | 12 (30%) |

Abbreviations: CD: Crohn’s disease; UC: ulcerative colitis; COPD: chronic obstructive pulmonary disease.

**Supplementary Table S2.** Treatment Satisfaction and Reasons of dissatisfaction Stratified by Disease Type

|  | **Total** | **CD** | | **UC** |
| --- | --- | --- | --- | --- |
| **Treatment satisfaction, n (%)** | **Ν=271** | **N=188** | | **Ν=83** |
| Not at all | 10 (3,7%) | 7 (3,7%) | | 3 (3,6%) |
| Little | 23 (8,5%) | 16 (8,5%) | | 7 (8,4%) |
| Quite | 73 (26,9%) | 55 (29,3%) | | 18 (21,7%) |
| A lot | 73 (26,9%) | 58 (30,9%) | | 15 (18,1%) |
| Very much | 92 (33,9%) | 52 (27,7%) | | 40 (48,2%) |
| **Reasons of dissatisfaction, n (%)** | **N=215** | **N=155** | | **N=60** |
| Side effects | 27 (12,6%) | | 19 (12,3%) | 8 (13,3%) |
| I have frequent stools | 32 (14,9%) | | 24 (15,5%) | 8 (13,3%) |
| I have frequent flares | 33 (15,3%) | | 19 (12,3%) | 14 (23,3%) |
| The frequency of doses | 13 (6%) | | 9 (5,8%) | 4 (6,7%) |
| The cost of medications | 4 (1,9%) | | 3 (1,9%) | 1 (1,7%) |
| I experience more abdominal pain | 9 (4,2%) | | 6 (3,9%) | 3 (5%) |
| Fatigue is increasing | 44 (20,5%) | | 32 (20,6%) | 12 (20%) |
| I do not like the mode of administration | 16 (7,4%) | | 13 (8,4%) | 3 (5%) |
| I experience more urgency to go to the bathroom | 14 (6,5%) | | 11 (7,1%) | 3 (5%) |
| Other reason | 23 (10,7%) | | 19 (12,3%) | 4 (6,7%) |

Abbreviations: CD: Crohn’s disease; UC: ulcerative colitis

**Supplementary Table S3.** Treatment Adherence and Reasons of Non-Adherence Stratified by Disease Type

|  | **All** | **CD** | **UC** |
| --- | --- | --- | --- |
| **Treatment adherence, n (%)** | **Ν=** **266** | **N=** **183** | **Ν=** **83** |
| I follow my treatment regularly | 237 (89,1%) | 174 (95,1%) | 63 (75,9%) |
| There are few times I forget to/ I do not take my treatment | 21 (7,9%) | 6 (3,3%) | 15 (18,1%) |
| Sometimes I forget to/ I do not take my treatment | 5 (1,9%) | 1 (0,5%) | 4 (4,8%) |
| Many times, I forget to/ I do not take my treatment | 3 (1,1%) | 2 (1,1%) | 1 (1,2%) |
| I never take my treatment | **-** | **-** | **-** |
| **Reasons of non-adherence, n (%)** | **N=34** | **N=13** | **N=21** |
| I feel that my symptoms are under control | 11 (32,4%) | 4 (30,8%) | 7 (33,3%) |
| Mode of administration | 2 (5,9%) | 1 (7,7%) | 1 (4,8%) |
| Frequent drug doses | 4 (11,8%) | 3 (23,1%) | 1 (4,8%) |
| The drug is not effective | 1 (2,9%) | 0 (0%) | 1 (4,8%) |
| Fear of side effects | 5 (14,7%) | 4 (30,8%) | 1 (4,8%) |
| Other reason | 11 (32,4%) | 1 (7,7%) | 10 (47,6%) |

Abbreviations: CD: Crohn’s disease; UC: ulcerative colitis

**Supplementary Table S4.** Factors associated with treatment satisfaction†: univariate and multivariate logistic regressions analyses

| **Satisfaction** | Univariate analysis | | Multivariate analysis | |
| --- | --- | --- | --- | --- |
|  | OR [95% CI] | p-value | OR [95% CI] | p-value |
| **Gender** |  |  |  |  |
| Male | Ref |  |  |  |
| Female | 0.873 (0.533 – 1.429) | 0.589 |  |  |
| **Age** |  |  |  |  |
| <50 years | Ref |  |  |  |
| 50 years or more | 0.969 (0.545 – 1.726) | 0.916 |  |  |
| **Employment status** |  |  |  |  |
| In paid employment | Ref |  | Ref |  |
| Other | 0.480 (0.270 – 0.852) | 0.012 | 0.561 (0.339 - 0.928) | 0.059 |
| **BMI** |  |  |  |  |
| Underweight and normal | Ref |  |  |  |
| Overweight and obese | 0.771 (0.467 – 1.272) | 0.308 |  |  |
| **Smoking status** |  |  |  |  |
| Never | Ref |  |  |  |
| Former smoker | 1.243 (0.659 – 2.345) | 0.501 |  |  |
| Current smoker | 0.703 (0.398 – 1.241) | 0.224 |  |  |
| **Disease activity** |  |  |  |  |
| Inactive* | Ref |  | Ref |  |
| Active** | 0.264 (0.154 – 0.453) | <0.001 | 0.288 (0.168 – 0.492) | <0.001 |
| **Age at diagnosis** |  |  |  |  |
| 0-30 years | Ref |  |  |  |
| >30 years | 0.746 (0.457 – 1.216) | 0.24 |  |  |
| **Disease duration** |  |  |  |  |
| <10 years | Ref |  |  |  |
| >10 years | 0.855 (0.523 – 1.397) | 0.532 |  |  |
| **Surgery** |  |  |  |  |
| No | Ref |  |  |  |
| Yes | 0.623 (0.337 – 1.152) | 0.161 |  |  |
| **Disease** |  |  |  |  |
| CD | Ref |  |  |  |
| UC | 1.393 (0.812 – 2.389) | 0.229 |  |  |
| **Comorbidities** |  |  |  |  |
| No | Ref |  |  |  |
| One or more | 0.651 (0.350 – 1.210) | 0.175 |  |  |

Abbreviations: BMI: body mass index; CI: confidence interval; OR: odds ratio; ref: reference value.

†Logistic regression: the dependent variable of satisfaction was categorized as 0: “not at all/little/quite satisfied” and 1: “a lot/very much satisfied”.

*Patients in remission.

** Patients with mild, moderate or severe disease activity.

**Supplementary Table S5.** Factors associated with treatment adherence†: univariate and multivariate logistic regressions analyses

| **Adherence** | Univariate analysis | | Multivariate analysis | |
| --- | --- | --- | --- | --- |
|  | OR [95% CI] | p-value | OR [95% CI] | p-value |
| **Gender** |  |  |  |  |
| Male | Ref |  | Ref |  |
| Female | 0.463 (0.226 – 0.947) | 0.077 | 0.399 (0.170 – 0.936) | 0.076 |
| **Age** |  |  |  |  |
| <50 years | Ref |  |  |  |
| 50 years or more | 0.793 (0.383 – 1.644) | 0.601 |  |  |
| **Employment status** |  |  |  |  |
| In paid employment | Ref |  |  |  |
| Other | 1.320 (0.544 – 3.203) | 0.539 |  |  |
| **BMI** |  |  |  |  |
| Underweight and normal | Ref |  |  |  |
| Overweight and obese | 1.214 (0.559 – 2.640) | 0.624 |  |  |
| **Smoking status** |  |  |  |  |
| Never | Ref |  |  |  |
| Former smoker | 0.874 (0.320 – 2.384) | 0.792 |  |  |
| Current smoker | 0.759 (0.304 – 1.894) | 0.554 |  |  |
| **Disease activity** |  |  |  |  |
| Inactive * | Ref |  |  |  |
| Active ** | 0.918 (0.420 – 2.008) | 0.831 |  |  |
| **Age at diagnosis** |  |  |  |  |
| 0-30 years | Ref |  |  |  |
| >30 years | 1.048 (0.483 – 2.275) | 0.905 |  |  |
| **Disease duration** |  |  |  |  |
| <10 years | Ref |  | Ref |  |
| > 10 years | 2.129 (0.963 – 4.704) | 0.062 | 1.980 (0.886 – 4.427) | 0.163 |
| **Surgery** |  |  |  |  |
| No | Ref |  | Ref |  |
| Yes | 5.120 (1.161 – 22.568) | 0.031 | 2.531 (0.674 – 9.511) | 0.248 |
| **Disease** |  |  |  |  |
| CD | Ref |  | Ref |  |
| UC | 0.163 (0.070 – 0.377) | <0.001 | 0.240 (0.106 – 0.546) | 0.004 |
| **Comorbidities** |  |  |  |  |
| No | Ref |  |  |  |
| One or more | 0.629 (0.236 – 1.675) | 0.353 |  |  |

Abbreviations: ΒΜΙ: body mass index; CI: confidence interval; OR: odds ratio; ref: reference value.

†Logistic regression: the dependent variable of adherence was categorized as 0: “I forget to/ I do not take my treatment (few/some/many times /always)” and 1: “I follow my treatment regularly”.

*Patients in remission.

** Patients with mild, moderate or severe disease activity.
